# Supplementary material for: ATNIVS biomarker heterogeneity in real-world patients receiving lecanemab
Source: J Prev Alzheimers Dis. 2026 Apr 11;13(6):100567. doi: 10.1016/j.tjpad.2026.100567 (PMC13092606; doi:10.1016/j.tjpad.2026.100567)
Supplement: Supplementary file 1 [file mmc1.docx]

|  | did not start lecanemab  (n = 15) | started lecanemab (n = 93) |
| --- | --- | --- |
| Age | 75.9 ±8.0 | 74.2 ±8.1 |
| Sex (female) | 56.2% | 73.1% |
| MCI | 37.5% | 62.4% |
| Global CDR 0.5 | 84.6% | 88.2% |
| CDR-SB | 2.65 ±1.33 | 2.99 ±1.31 |
| MMSE | 25.1 ±2.1 | 24.7 ±2.0 |
| *APOE* ε4 carrier | 22.2% (2/9) | 32.4% (12/37) |
| PET centiloid  mean | 55.1 ±21.1 | 60.1 ±24.5 |
| range | 20.8 to 77.9 | 3.1 to 112 |

**Supplementary Table 1**. Baseline characteristics of the amyloid-positive patients who did not start lecanemab

The reasons for not starting lecanemab were contraindication on MRI (n = 7), CDR global score 0 (n = 2), opt out (n = 5), and selection of donanemab that became available (n = 1).

Abbreviations

CDR, clinical dementia rating; CDR-SB, CDR sum of boxes; MCI, mild cognitive impairment; MMSE, mini-mental state examination; PET, positron-emission tomography

|  | Clinical < Biological (n =5) | Concordant (n = 3) | Clinical > Biological (n = 3) |
| --- | --- | --- | --- |
| Age | 70.8 ±8.5 | 71.3 ±10.7 | 82.0 ±3.0 |
| Female | 80% | 100% | 66.7% |
| plasma NfL | 16.6 ±0.9 | 68.4 ±49.4 | 31.3 ±8.9 |
| plasma GFAP | 278 ±8.3 | 355 ±267 | 322 ±216 |
| Fazekas DWM | 1 [0–1] | 1 [1–1] | 2 [1.5–2] |
| Fazekas PV | 1 [1–1] | 1 [0.5–1] | 2 [2–2.5] |

**Supplementary Table 2**. Comparison between groups based on the association between clinical stage and biological stage based on tau PET [6]

There were some trends, including higher age and Fazekas scores in the “Clinical > Biological” group and lower plasma NfL in the “Clinical < Biological” group. However, the sample size was very small, and the difference did not reach statistical significance.

Abbreviations

DWM, deep white matter; GFAP, glial fibrillary acidic protein; NfL, neurofilament light chain; PV, periventricular
